# Association of Angiopoietin Dysregulation in Placental Malaria with Adverse Birth Outcomes

**DOI:** 10.1155/2020/6163487

**Published:** 2020-01-13

**Authors:** Puspendra P. Singh, Sneha Bhandari, Ravendra K. Sharma, Neeru Singh, Praveen K. Bharti

**Affiliations:** ^1^National Institute of Research in Tribal Health (NIRTH), 482003, Jabalpur, Madhya Pradesh, India; ^2^Department of Biological Science, University of Notre Dame, South Bend IN-46556, USA

## Abstract

Malaria in pregnancy causes adverse birth outcomes due to sequestration of *Plasmodium falciparum*-infected erythrocytes in the placenta. Angiopoietins are critical regulators of vascular development and formation of placental villous vasculature. Angiopoietin-1 and Angiopoietin-2 concentrations were measured in peripheral and placental plasma samples from 70 malaria-infected and 216 control women using commercially available DuoSet ELISA development kit. Angiopoietins increased in placental plasma (ANG1-5833.5 pg/ml and ANG2-9580.6 pg/ml) as compared to peripheral plasma (ANG1-2293.1 pg/ml and ANG2-1198.9 pg/ml, *p* < 0.0001). The concentration of placental and peripheral ANG1 (6099.23 pg/ml and 2320.5 pg/ml) was significantly lower (5013.5 pg/ml, 2208.5 pg/ml), and ANG2 (9553.3 pg/ml, 1180.92 pg/ml) was significantly higher (9664.6 pg/ml, 1254.4 pg/ml) in malaria-positive cases as compared to malaria-negative (*p* < 0.0001). The association of dysregulated angiopoietins in malaria with adverse birth outcomes showed that the peripheral and placental ANG1 concentration was lower and ANG2 concentration was higher in low-birth-weight baby and stillbirth birth outcome as compared to normal deliveries among malaria-positive group. Therefore, ANG1 and ANG2 could be considered a biomarker for adverse outcome during malaria in pregnancy.

## 1. Introduction

*Plasmodium falciparum* infection can cause significant adverse outcomes to mothers and babies. The severity of these consequences depends upon the transmission intensity of the region as it influences the immunity to malaria. In areas with intense transmission of malaria, low birth weight (LBW) and maternal anemia are the major severe outcomes associated with maternal malaria. On the contrary, in low transmission areas, *P. falciparum* infection can contribute to spontaneous abortion, preterm delivery, intrauterine growth restriction (IUGR), fetal death, and severe malaria in pregnant women [1–3]. Therefore, studies in different endemic regions are needed to better understand the pathogenic mechanisms and risk factors associated with malaria during pregnancy.

Since *P. falciparum* parasites sequester in large numbers in the placenta, intense efforts have been made to understand the sequestration mechanisms, histological changes, and immunological process in the infected placenta [4]. Despite these efforts, biological mechanisms contributing to low-birth-weight babies, preterm delivery, and stillbirth (SB) in malaria-infected women are not well understood.

Angiopoietins (ANG1 and ANG2) play a vital role in the vascular development [5]. ANG1 and ANG2 function through a common receptor called Tie-2, and alterations in their ratio will have important consequences for their function [6]. Typically, when ANG1 levels are higher than ANG2, it helps to maintain normal vasculature. When the ANG2 level is higher than ANG1, it leads to vascular development [7]. ANG1 and ANG2 have been found to be expressed in placental syncytiotrophoblasts and cytotrophoblasts which are important components of placental villi [8]. Sequestration of *P. falciparum* parasites in the placental intervillous blood can potentially alter the relative levels of Ang1/Ang2 and contribute to their functional imbalance [9, 10]. A study reports that ANG1 and ANG2 levels were altered in *P. falciparum*-infected mothers from Africa in favour of a higher ANG2/ANG1 ratio. It was also demonstrated that this dysregulation was associated with low birth weight in malaria-infected women [11]. Therefore, it is important to understand the reproducibility of these findings in Asia where the epidemiology of malaria and host immunity is distinct from a typical African malaria setting. We hypothesized that *P. falciparum* malaria infection in pregnant women in an Indian setting may lead to dysregulation of angiopoietins and this may be associated with low birth weight and stillbirth. Therefore, we investigated the role of Ang1 and Ang2 in influencing the adverse outcomes associated with *P. falciparum* infection in pregnant women residing in a low transmission setting in India.

## 2. Materials and Methods

### 2.1. Study Site and Sampling Method

This study has been carried out during July-2010 to Dec-2012 at the delivery unit at Civil Hospital Maihar, which is a secondary health facility and caters to health needs of rural, semiurban, and ethnic tribal population. Pregnant women who presented at the delivery unit were screened for malaria by microscopic examination of peripheral blood smears. This study had been approved by Institutional Ethics Committee. The women whose peripheral and placental samples were malaria-negative/positive were considered as placental malaria-negative (PM-)/placental malaria-positive (PM+) group, respectively. The numbers of samples for PM- and PM+ groups were chosen on the premise of roughly the same percentage of low-birth-weight baby (LBW), stillbirth (SB), and gravidity in each group. Written informed consent was taken from pregnant women at the time of enrolment for the study.

### 2.2. Sample Collection

At the time of enrollment, socioeconomic and clinical data (fever history, clinical symptoms, hemoglobin, and axillary temperature) were recorded. Peripheral blood smears (thick and thin) were prepared for malaria parasite examination, and about 1 ml of peripheral blood was collected for immunological study. After delivery, the expelled placenta was taken for malaria examination and for intervillous blood (incision method) collection. Plasma was separated from all blood samples by centrifugation at 3000 rpm for 10 minutes and stored in -80°C until used for experiments.

### 2.3. Birth Outcomes

A baby born with no sign of life at or after 28 weeks of gestation was considered a stillbirth. Live birth was defined as complete expulsion of a live baby, irrespective of the duration of the pregnancy. Babies with birth weight below 2500 grams were considered LBW babies, and other babies were considered to have normal birth weight (NBW). Note that birth weight was not measured for stillbirths.

### 2.4. Measurement of Angiopoietin Levels in Plasma

The peripheral and placental intervillous blood (IVB) plasma levels of ANG1 and ANG2 were measured using the commercially available DuoSet ELISA development kits from R&D systems, according to the manufacturer's instructions. Plasma samples were diluted 1 : 4 and 1 : 2 in the assay buffer for ANG1 and ANG2, respectively.

### 2.5. Statistical Analysis

Statistical analysis was performed using STATA. Categorical variables were analysed using the chi-squared test, and continuous variables were analysed using the Student *t*-test and ANOVA test to examine the association of angiopoietins in birth outcome and adverse birth outcomes among malaria-positive and malaria-negative cases. ROC curve and area under curve (AUC) were also computed to examine the sensitivity and specificity by ANG1 and ANG2 level to discriminate between PM+ and PM- cases.

## 3. Results

A total of 7873 women were screened, and women over the age of 18 with no chronic disease were asked to participate in the study. By microscopy, 101 women were found to have *P. falciparum* infection in both placental and peripheral blood and out of these, 70 were included in the study as placental malaria-positive cases (PM+), the rest of the 31 women were excluded due to missing data either maternal characteristics or birth outcomes. A subset of 216 women (1 : 3 ratio) whose peripheral and placental smears were negative were considered as placental malaria negative (PM- control group). As summarized in Table 1, PM+ women had a significantly higher body temperature, lower hemoglobin, and lower baby weight as compared to PM- women. Because the samples of malaria-negative and malaria-positive groups were taken as matched in terms of gravidity and birth outcomes, these variables did not and were not expected to have any significant difference among these groups. Peripheral and placental ANG1 level was significantly lower (*p* < 0.0001) and ANG2 was significantly higher (*p* < 0.0001) in PM+ women as compared to PM- women (Table 1).

### 3.1. Relationship of Angiopoietins with Birth Outcomes

ANG1 was lower in mothers delivering a LBW baby (2291.1 pg/ml) and SB (2231.2 pg/ml) birth outcome as compared to normal birth (2330.8 pg/ml) while ANG2 level was higher in LBW (1197.9 pg/ml) and SB (1225.8 pg/ml) birth outcome as compared to normal birth (1184.4 pg/ml) in peripheral plasma. In placental plasma, same pattern were found, i.e., ANG1 was lower in LBW (5780.9 pg/ml) and SB (5400.8 pg/ml) birth outcome as compared to normal birth (6135.6 pg/ml) and ANG2 was higher in LBW (9584.3 pg/ml) and SB (9624.8 pg/ml) birth outcome as compared to normal (9551.4 pg/ml) birth. ANOVA analysis was carried out to test the significant association, and the test revealed that lower concentration of peripheral and placental ANG1 protein (*p* < 0.0001, *p* < 0.0001, respectively) and higher concentration of peripheral and placental ANG2 protein (*p* < 0.001) were significantly associated with SB birth outcome as compared to normal birth outcome except in the case of peripheral ANG2 for mothers with low birth weight (Table 2). The ANOVA analysis also reveals that the ratio of peripheral ANG1 and ANG2 and ratio of placental ANG1 and ANG2 also vary considerably among normal, low birth weight, and stillbirth. Ratios of peripheral ANG1/ANG2 and placental ANG1/ANG2 were significantly higher in LBW and SB birth categories.

### 3.2. Level of Angiopoietin among Birth Outcome within Malaria Status

We explored the angiopoietin dysregulation and their association with PM and birth outcomes. The peripheral ANG1 level was significantly lower in normal birth (2276.1 ± 58.8*vs*2349.2 ± 139.8), low birth weight (2222.9 ± 81.6*vs*2310.9 ± 159.8), and stillbirth (2075.9 ± 37.3*vs*2287.3 ± 71.4) in the PM+ group compared to the PM- group. Similarly, the placental ANG1 level was significantly lower in the PM+ group compared to the PM- group in all three birth outcomes. But, on contrast, the peripheral ANG2 level was significantly higher in PM+ compared to PM- in normal birth (1216.3 ± 70.0*vs*1173.6 ± 69.7), low birth weight (1258.7 ± 53.2*vs*1180.3 ± 77.6), and stillbirth (1310.7 ± 24.2*vs*1195.1 ± 29.6). Similarly, in case of placental plasma values, ANG2 was higher in PM+ group compared to PM- groups in all birth outcomes categories (Table 3). Statistical analysis to test the significant association of angiopoietin dysregulation in birth outcome category among PM- and PM+ group revealed that the peripheral ANG1 level in the PM+ group was significantly lower in LBW (2222.9 ± 81.6*vs*2276.1 ± 58.8) and SB birth (2075.9 ± 37.3*vs*2276.1 ± 58.8) outcome as compared to normal, whereas a high level of peripheral ANG2 in PM+ was seen in LBW (1258.7 ± 53.2*vs*1216.3 ± 70.0) and SB birth outcome (1310.7 ± 24.2*vs*1216.3 ± 70.0) as compared to normal birth outcomes. The angiopoietin level of placental plasma in the PM+ group also showed the same pattern (i.e., a significant decrease of ANG1 and increase of ANG2) in adverse birth outcome as compared to normal. The ratio of peripheral (AGN2/AGN1) was statistically higher in the PM+ group compared to the PM- group within the birth outcome categories and among PM+ cases in LBW (0.57 ± 0.03*vs*0.53 ± 0.03) and SB (0.63 ± 0.02*vs*0.53 ± 0.03) compared to normal birth outcome. Similarly, the ratio of placental (AGN2/AGN1) was statistically higher in the PM+ group compared to the PM- group within the birth outcome categories and among PM+ cases in LBW (1.99 ± 0.11*vs*1.75 ± 0.28) and SB (0.2.34 ± 0.23*vs*1.75 ± 0.28) compared to normal birth outcome (Table 3).

**Figure 1:**
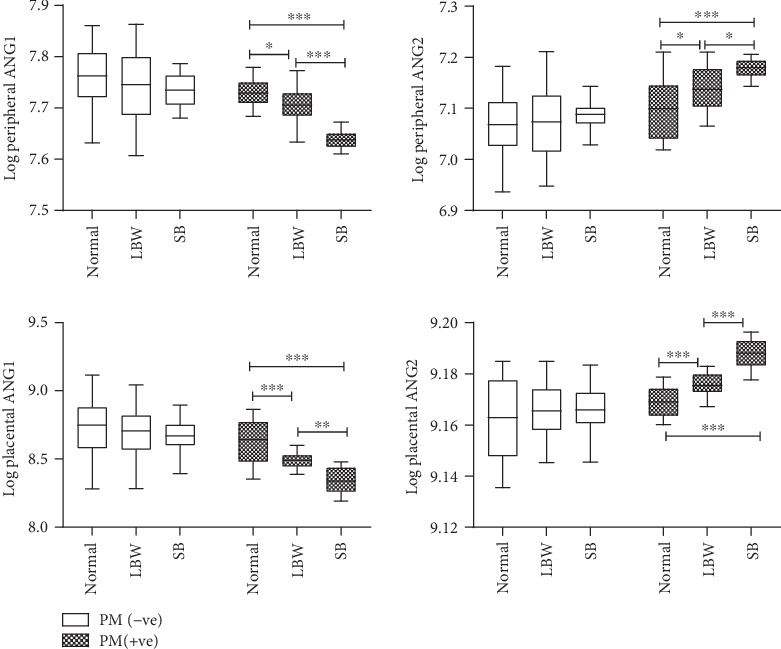
Angiopoietin level in birth outcome category among the PM- and PM+ groups. Box plot shows the median, interquartile range with whiskers denoting the maximum and minimum values. ^∗^*p* < 0.02, ^∗∗^*p* < 0.002, ^∗∗∗^p < 0.0001.

Statistical analysis to test the significant association of angiopoietin dysregulation in birth outcome category among the PM- and PM+ groups revealed that the peripheral ANG1 level in the PM+ group was significantly lower in LBW (*p* < 0.02) and SB (*p* < 0.0001) birth outcome as compared to normal. A high level of peripheral ANG2 in PM+ was seen in LBW (*p* < 0.02) and SB (*p* < 0.0001) birth outcome as compared to normal birth outcomes. The angiopoietin level of placental plasma in the PM+ group also showed the same pattern (i.e., a significant decrease of ANG1 and increase of ANG2) in adverse birth outcome as compared to normal (Figure 1).

**Figure 2:**
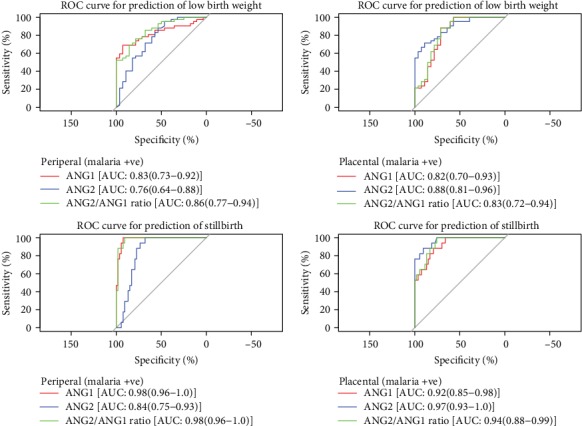
ROC curve of peripheral and placental angiopoietin levels in birth outcome category among malaria-positive mothers.

A receiver operating characteristic (ROC) curve was drawn to predict the sensitivity and specificity of ANG1 and ANG2 levels with birth outcome in malaria-positive mothers. The ROC curve and area under the curve show that all variables in peripheral ANG1 and ANG2, placental ANG1 and ANG2, and ratio of peripheral (ANG2/ANG1) and placental (ANG2/ANG1) show a significantly higher area than the hypothetical area under curve 0.5. Area under curve of ratio of ANG2/ANG1 was 0.86 in the case of peripheral and 0.83 in placental malaria-positive mothers in case of LBW. Similarly, in the case of SB ratio, ANG2/ANG1 was 0.98 in peripheral and 0.94 in placental malaria-positive mothers (Figure 2).

## 4. Discussion

Human pregnancy is characterized by angiogenesis, tissue development, and remodeling [12, 13]. The disturbance in the balance of proangiogenic and antiangiogenic factors can lead to impaired placentation, causing major pregnancy complications such as preeclampsia and intrauterine growth restriction which can lead to poor obstetric outcomes [14]. Human parturition has a common pathway characterized by increased uterine contractility, cervical ripening/dilation, and membrane/decidual activation, culminating in membrane rupture [15]. This activation is a coordinated inflammatory phenomenon in normal spontaneous labor during the term delivery [16, 17]. This hypothesis is also proven in a mouse model as increased expression of angiogenesis-related genes in mouse uterus was observed in spontaneous labor at term delivery as well as pathologically induced preterm labor [18].

In the present study, the angiopoietin level was significantly elevated in the placental plasma as compared to the peripheral plasma which supports the previous findings that endometrium, deciduas, and placenta are rich sources of angiogenic growth factor [19, 20]. Many studies reviewed by Zygmunt et al. showed that angiopoietins play an important role for successful placentation as well as parturition [21]. Placental vascular development and remodeling are controlled primarily through the highly regulated actions of angiogenic factors from the VEGF and angiopoietin families [12]. ANG1 has a major role in the stabilization and maturation of newly formed vessels, whereas ANG2 provides the destabilizing effect necessary to initiate vascular remodeling [22, 23]. The downregulation of ANG1 and upregulation of ANG2 in *P. falciparum*-infected cases from the present study are very much similar to previous studies [11, 24]. The angiogenic factor perturbation has also been reported in preeclampsia and fetal growth restriction associated with inadequate placental vascularization [25]. Normally, the ANG1 level increases and ANG2 level decreases throughout the gestation but alterations in the levels of angiopoietins may cause pathological pregnancies [12, 13, 26]. Some studies related to association of angiopoietins in cerebral malaria (CM) demonstrated that ANG1 act as a good biomarker in differentiating CM from mild malaria among Thai adults, but not in Ugandan children [27]. Yeo et al. reported that ANG2 is a better marker of severe malaria-associated deaths than lactate in Indonesian adults [10]. Among Ugandan children, higher levels of ANG1 were found to be associated with a reduced risk of death [27]. In a recent study conducted with Malawian children, ANG1 levels were significantly downregulated among CM patients with retinopathy compared to those without retinopathy, uncomplicated malaria patients, and those with nonmalarial encephalopathy [28]. Involvement of ANG1 and ANG2 in the pathogenesis of cerebral malaria was also studied in Central India and revealed that the concentration of ANG1 was lower in the cerebral malaria survivor and nonsurvivor groups as compared to mild malaria and healthy control. ANG2 showed positive association with malaria severity [29].

Consistent with earlier studies, the present study highlights that the altered level of angiopoietin due to malaria (infection/inflammation) may be associated with the pathogenesis of birth outcomes. Placental malaria (PM) is believed to lead to placental insufficiency, a progressive deterioration in placental function and inability to sustain fetal growth, resulting in LBW infants and also increased risk of perinatal mortality [30]. Previous reports are consistent with the hypothesis that PM may influence angiogenesis and vascular development. Histological and ultrasound studies of PM suggest that malaria infection alters the placental vascular structure [31, 32]. Altered levels of angiopoietins, sEng, VEGF, sFlt-1, or C5a have recently been shown in several populations of African women to have with PM at delivery [11, 24, 33]. Although the mechanism of adverse birth outcomes (LBW and stillbirth) is poorly understood, it is hypothesized that these pregnancy outcomes may result of intrauterine growth restriction and/or premature delivery [34]. Both IUGR and spontaneous premature delivery can result from functional placental insufficiency where nutrient supply is inadequate to support fetal growth and continued in utero-development [35].

The mean concentration of ANG1 was decreased, and ANG2 was increased in women with malaria who delivered LBW and stillbirth baby in the current study. These findings are in line with those earlier reports that support that the dysregulation of angiopoietins was associated with complicated pregnancy outcome [36]. Low ANG1 was reported to lead to vessel destabilization and a decrease in the angiogenic sprouting promoting vessel leakage [37] which may be the explanation of adverse birth outcome. Several scenarios have been proposed to account for the serum measurement of angiogenic factors in maternal blood according to early pregnancy state. Involvement of angiogenic factors in miscarriage, abnormal placentation, preeclampsia, and IUGR pregnancies was also shown in various studies [38]. The study of Conroy et al. also suggested that activation of complement system (C5a) due to malaria caused dysregulation of angiogenic factor, i.e., ANG1 was negatively and ANG2 was positively associated with complement activation which was found to be associated with fetal growth restrictions [39].

The finding of this present study is consistent with few published studies which can lead to established ANG1 and ANG2 as biomarker for the severity due to malaria during pregnancy.

## Figures and Tables

**Table 1:** Mother's characteristics and birth outcomes by placental malaria status.

	Malaria (-ve) *N* = 216	Malaria (+ve) *N* = 70	*p* value^∗∗^
Characteristics of mother			
Age^a^	24.1 ± 3.8	24.7 ± 4.0	0.27
Axillary temperature (°C)^a^	36.0 ± 1.4	36.7 ± 1.3	0.002
Haemoglobin (g/dl)^a^	11.2 ± 2.6	09.6 ± 2.2	<0.0001
Anaemia	89 (41%)	49 (70%)	<0.0001
Gravidae^∗^			
Primgravid	72 (33.3%)	25 (35.7%)	
Secundigravid	71 (32.9%)	22 (31.4%)	
Multigravid	73 (33.8%)	23 (32.9%)	
Angiopoietin (pg/ml)^a^			
Peripheral ANG1	2320.5 ± 138.7	2208.5 ± 101.3	<0.0001
Peripheral ANG2	1180.9 ± 67.0	1254.4 ± 66.5	<0.0001
Placental ANG1	6099 ± 1177.9	5013.6 ± 831.2	<0.0001
Placental ANG2	9553.3 ± 119.0	9664.6 ± 88.2	<0.0001
Birth outcomes			
Normal^∗^	83 (38.4%)	28 (40.0%)	
Low birth weight^∗^	86 (39.8%)	25 (35.7%)	
Stillbirth^∗^	47 (21.8%)	17 (24.3%)	
Baby weight (gm)^a#^	2470.4 ± 382.0	2292.8 ± 442.8	0.005

^a^Mean ± SD; ^∗^indicates variable used to frequency match PM+ and PM- groups; ^∗∗^*p* values reported are Pearson chi-square test for categorical variable and two sample *t*-test for continuous variables; ^**#**^Birth weight information was collected for 53 babies born from PM+ and 169 babies born from PM- women.

**Table 2:** Angiopoietin concentration (pg/ml) among birth outcome category.

Birth outcomes	Mean ± SD					
	Peripheral ANG1	Peripheral ANG2	Placental ANG1	Placental ANG2	Ratio of peripheral ANG2/ANG1	Ratio of placental ANG2/ANG1
Normal (111)	2330.8 ± 128.2	1184.4 ± 71.9	6135.6 ± 1269.4	9551.4 ± 131.8	0.510 ± 0.040	1.624 ± 0.340
LBW (111)	2291.1^∗^ ± 150.2	1197.9 ± 79.8	5780.9^∗^ ± 1165.6	9584.3^∗^ ± 102.1	0.526^∗^ ± 0.054	1.724^∗^ ± 0.338
SB (64)	2231.2^∗∗^ ± 113.7	1225.8^∗∗^ ± 58.6	5400.8^∗∗^ ± 969.0	9624.8^∗∗^ ± 123.4	0.552^∗∗^ ± 0.052	1.846^∗∗^ ± 0.370
Total (286)	2293.1 ± 139.0	1198.9 ± 73.9	5833.5 ± 1196.9	9580.6 ± 121.9	0.525 ± 0.051	1.713 ± 0.355
F-statistics (p)	11.183 *p* ≤ 0.0001	6.663 *p* ≤ 0.001	8.221 *p* ≤ 0.0001	7.783 *p* ≤ 0.001	15.549 *p* ≤ 0.0001	8.422 *p* ≤ 0.0001

Note: Level of significance between normal and LBW/SB based on *t*-test, ^∗∗^*p* ≤ 0.01, ^∗^*p* ≤ 0.05.

**Table 3:** Angiopoietin concentration (pg/ml) in birth outcome category with malaria status.

Birth outcome	Malaria result	Mean ± SD					
		Peripheral ANG1	Peripheral ANG2	Placental ANG1	Placental ANG2	Ratio of peripheral ANG2/ANG1	Ratio of placental ANG2/ANG1
Normal	Negative (83)	2349.2 ± 139.8	1173.6 ± 69.7	6307.5 ± 1335.0	9536.1 ± 146.1	0.50 ± 0.04	1.58 ± 0.35
	Positive (28)	2276.1^∗∗^ ± 58.8	1216.3^∗∗^ ± 70.0	5626.1^∗∗^ ± 890.0	9596.7^∗∗^ ± 55.4	0.53^∗∗^ ± 0.03	1.75^∗^ ± 0.28
LBW	Negative (86)	2310.9 ± 159.8	1180.3 ± 77.6	6045.7 ± 1192.1	9561.5 ± 103.0	0.51 ± 0.05	1.65 ± 0.34
	Positive (25)	2222.9^∗∗^ ± 81.6	1258.7^∗∗^ ± 53.2	4869.9^∗∗^ ± 280.9	9662.9^∗∗^ ± 43.7	0.57^∗∗^ ± 0.03	1.99^∗∗^ ± 0.11
SB	Negative (47)	2287.3 ± 71.4	1195.1 ± 29.6	5829.4 ± 723.9	9568.9 ± 87.3	0.52 ± 0.02	1.67 ± 0.22
	Positive (17)	2075.9^∗∗^ ± 37.3	1310.7^∗∗^ ± 24.2	4216.0^∗∗^ ± 399.7	9779.2^∗∗^ ± 59.3	0.63^∗∗^ ± 0.02	2.34^∗∗^ ± 0.23
Total	Negative (216)	2320.5 ± 138.7	1180.9 ± 67.0	6099.2 ± 1177.9	9553.3 ± 119.0	0.51 ± 0.04	1.63 ± 0.32
	Positive (70)	2208.5^∗∗^ ± 101.3	1254.4^∗∗^ ± 66.5	5013.6^∗∗^ ± 831.2	9664.7^∗∗^ ± 88.2	0.57^∗∗^ ± 0.05	1.98^∗∗^ ± 0.32
Grand total (286)		2293.1 ± 139.0	1198.9 ± 73.9	5833.5 ± 1196.9	9580.6 ± 121.9	0.53 ± 0.05	1.71 ± 0.35

Note: Level of significance between negative and positive cases based on *t*-test, ^∗∗^*p* ≤ 0.01, ^∗^*p* ≤ 0.05.

## Data Availability

We have reported all the findings in the manuscript. The patient information sheets are available with the corresponding author.
